# Circulating follicular helper T cells are associated with mortality in sepsis

**DOI:** 10.3389/fimmu.2026.1931433

**Published:** 2026-10-28

**Authors:** Yingqian Sun, Xinling Pan, Chengliang Zhang, Le Kang, Yuan Li, Longyi Zhang

**Affiliations:** 1 Clinical Laboratory, Affiliated Dongyang Hospital of Wenzhou Medical University, Dongyang, Zhejiang, China; 2 Department of Biomedical Sciences Laboratory, Affiliated Dongyang Hospital of Wenzhou Medical University, Dongyang, Zhejiang, China

**Keywords:** B cells, CD4^+^ T cells, follicular helper T cells, follicular regulatory T cells, sepsis

## Abstract

**Introduction:**

B-cell dysfunction is a key component of immune derangement in sepsis. Follicular helper T (Tfh) cells and follicular regulatory T (Tfr) cells play critical roles in B cell maturation and differentiation. The aim of this study was to investigate whether the balance of circulating Tfh subsets (Tfh1, Tfh2, Tfh17, and Tfh17.1) and Tfr cells is altered in sepsis and evaluate their associations with B cell subsets and 28-day mortality.

**Methods:**

Peripheral blood Tfh, Tfr, and B cell subsets were measured by flow cytometry in 69 healthy controls and 123 patients with sepsis on day 1 and reassessed on day 7.

**Results:**

Compared with healthy controls, patients with sepsis showed significant reductions in the numbers of Tfh cell subsets and Tfr cells. Tfh subset composition was altered, with a decreased proportion of Tfh17.1 cells and a relatively increased proportion of Tfh17 cells. Tfr frequency was significantly lower, and Tfh/Tfr ratio was markedly elevated. Prognostic analysis showed that non-survivors had a lower Tfh cell percentage than survivors on day 1. Tfh percentage ≥ 17.05% was independently associated with lower 28-day mortality (OR [95% CI]: 0.331 [0.127, 0.861], *P* = 0.023). By day 7, absolute counts of Tfh cell subsets and Tfr cells were significantly lower in non-survivors than in survivors. Spearman’s partial correlation analysis indicated that the numbers of Tfh cell subsets and Tfr cells were positively correlated with memory or CD5^+^ B cells, except for Tfh1.

**Discussion:**

Tfh cell subsets in sepsis are redistributed, with an elevated Tfh/Tfr ratio. A Tfh (%) ≥ 17.05% was independently associated with lower 28-day mortality. These findings provide new insights into immune dysregulation in sepsis.

## 1 Introduction

Sepsis is defined as life-threatening organ dysfunction caused by a dysregulated host response to infection (1), accounting for approximately one-fifth of global deaths (2). Immune dysfunction is an important factor affecting the prognosis of sepsis (3), particularly immunosuppression, which leaves patients unable to clear the primary infection and more susceptible to secondary infections; this immunosuppressive stage accounts for most sepsis-related deaths (4). Global lymphopenia, affecting both B and T lymphocytes, is an important cause of immunosuppression in sepsis (5).

Humoral immunity is a critical component of host defense against pathogens. Decreased numbers of B cells and their subsets (6–8), as well as reduced immunoglobulin levels (9–11), have been associated with increased sepsis mortality.

Follicular helper T (Tfh) cells are a specialized subset of helper T cells involved in humoral immunity and closely interact with B cells. Antigen presentation by B cells is important for Tfh differentiation (12). In turn, Tfh cells promote B cell proliferation and differentiation, facilitating somatic hypermutation, antibody class switching, and the development of long-lived, high-affinity antibody-secreting cells or memory B cells within the germinal center (GC) (13). Without Tfh cells, B cells primarily produce low-affinity antibodies, such as those from short-lived plasma cells (14).

Circulating Tfh cells in human blood can be classified into four subsets based on the differential expression of CCR6 and CXCR3: Tfh1, Tfh2, Tfh17, and Tfh17.1 (15, 16). Tfh cell subsets characteristically produce IL-21 (17), a potent inducer of GC B cell proliferation, differentiation, and affinity maturation, and a promoter of IgG class switching (18). Each subset also exhibits distinct cytokine secretion profiles and B cell helper functions (19–21). The functional heterogeneity of Tfh subsets contributes to the production of different antibody classes. Tfh1 cells are primarily involved in antiviral immunity and responses to intracellular pathogens, promoting IgG subclass production (22). Tfh2 cells are associated with IgE/IgG4 class switching and responses to allergens and parasitic infections (18, 23). Tfh17 cells support the generation of IgG2a/IgG3 (24) and IgA (17). Tfh17.1 is a recently identified subset (25, 26) that shares features of both Tfh1 and Tfh17 (27), although its precise functional mechanisms remain unclear. Specific Tfh subsets may be required for effective B cell support during infections caused by different pathogens (28). For instance, in COVID-19 convalescent patients, elevated counts of Tfh1 and Tfh2 cells correlate with high serum neutralizing activity (29), whereas Tfh2 cells have been reported to impede humoral responses in chronic hepatitis B infection (30). Increased Tfh17 cell frequency is associated with enhanced specific antibody production following Ebola virus vaccination (31).

Follicular regulatory T (Tfr) cells are believed to regulate GC responses by suppressing excessive Tfh and B cell activation and proliferation. This regulation promotes the selection of high-affinity clones and inhibits autoreactive B cell clones, thereby optimizing affinity maturation (21, 32). The balance between Tfh and Tfr cells is critical for establishing effective humoral immunity while limiting autoimmunity (33).

Studies have shown that, compared with healthy controls (HCs), patients with sepsis exhibit reduced cellular density in secondary lymphoid organs, including decreased numbers of follicular B cells and Tfh cells (34). Reduced circulating Tfh cell percentage correlates with decreased B cell percentage and poor prognosis in sepsis (7), supporting the hypothesis that humoral immune dysregulation results from insufficient Tfh help. However, it remains unclear whether the balance of Tfh cell subsets is disrupted and whether the Tfh/Tfr ratio is altered in sepsis (28). Therefore, the aim of this study was to assess the correlation between these cell populations and B cell subsets, as well as patient prognosis, to improve understanding of humoral immune dysregulation in sepsis.

## 2 Materials and methods

### 2.1 Study population

This prospective cohort study was conducted at a tertiary general hospital (Dongyang Hospital Affiliated with Wenzhou Medical University) in China between March 2021 and May 2024. Patients were recruited from the emergency department or intensive care unit.

Approval was obtained from the Ethics Committee of the Affiliated Dongyang Hospital of Wenzhou Medical University (approval no.: 2021-YX-143), and all participants or their legal representatives provided written informed consent. The study was conducted in accordance with the Declaration of Helsinki.

Patients diagnosed with sepsis or septic shock according to the Sepsis-3.0 criteria (1) were enrolled within 24 h of admission. The exclusion criteria were as follows: (1) age < 18 years; (2) pregnancy or lactation; (3) use of antibiotics for > 2 days; (4) immune system diseases, malignant tumors, hematological disorders, human immunodeficiency virus infection, or chronic viral hepatitis; (5) immunosuppressive therapy; and (6) refusal to provide informed consent or loss to follow-up. In addition, 69 age- and sex-matched HCs were recruited from individuals undergoing routine health examinations. These participants were in good health and free from inflammatory diseases, and the same exclusion criteria were applied to them.

### 2.2 Data collection

Clinical data on day 1, including demographic characteristics, primary diagnoses, laboratory results, and Acute Physiology and Chronic Health Evaluation II (APACHE II) and Sequential Organ Failure Assessment (SOFA) scores, were collected from the clinical information system. Among the laboratory tests on admission, procalcitonin (PCT) was unavailable in one case (1.23%) and lactate (Lac) in two cases (2.47%) in the survivors. These missing values were imputed using the median values of the respective variables from the survivors. Microbiological data were obtained from the clinical laboratory. Twenty-eight-day all-cause mortality was determined using the clinical information system or follow-up telephone interviews.

### 2.3 Sample collection and flow cytometry

Fresh EDTA-anticoagulated blood samples (3 mL) were collected on day 1 (≤24 h). If patients remained hospitalized on day 7, an additional blood sample was collected. Lymphocyte counts were determined using a hematology analyzer (XN-9000; Sysmex, Japan).

Samples were acquired within 24 h of collection on a Navios^®^ flow cytometer (Beckman Coulter, Brea, CA, USA) to quantify the relative proportions of immune cell subsets; data were analyzed using Kaluza software (version 2.0; Beckman Coulter). Surface staining was performed using antibodies listed in **Supplementary Tables 1–3**, and the staining procedure is described in **Supplementary Figure 1**. Gating strategy 1 for Tfh cell analysis is illustrated in **Supplementary Figure 2**. Helper T cells were defined as CD4^+^ cells and classified into naïve (CD45RO^−^CCR7^+^), central memory (CD45RO^+^CCR7^+^), effector memory (CD45RO^+^CCR7^–^), and terminally differentiated effector memory cells re-expressing CD45RA (CD45RO^–^CCR7^–^) based on CD45RO and CCR7 expression. Tfh cells were defined as CXCR5+ cells among non-naïve T cells. Based on CCR6 and CXCR3 expression, Tfh subsets were identified as Tfh1 (CXCR3^+^CCR6^−^), Tfh2 (CXCR3^−^CCR6^−^), Tfh17 (CXCR3^−^CCR6^+^), and Tfh17.1 (CXCR3^+^CCR6^+^). Gating strategy 2 for identifying Tfr cells and the Tfh/Tfr ratios is illustrated in **Supplementary Figure 3**. Tfr cells were defined as (CD4^+^CXCR5^+^CD45RA^-^CD25^+^CD127^−/low^), and Tfh cells as (CD4^+^CXCR5^+^CD45RA^−^CD25^−^CD127^+^). The Tfh/Tfr ratio was calculated accordingly. Gating strategy 3 was used to identify circulating T lymphocytes and CD4^+^ T cells. Total T lymphocytes were identified by CD3 expression. Based on the expression of CD4/CD8, CD4^+^ T cells were defined as CD4^+^CD8^−^ (**Supplementary Figure 4**). The absolute counts of CD4^+^ T cells were calculated by multiplying the absolute lymphocyte count by the relevant proportions: CD4^+^ T = absolute lymphocyte count × (the proportion of CD3^+^ T cells) × (the proportion of CD4^+^ T cells). Then, based on the proportions of Tfh cells and their subsets obtained from gating strategy 1, and of Tfr cells from gating strategy 2, their corresponding absolute counts were calculated. The gating strategy for B cell subsets, including transitional (Tr), naïve, unswitched memory, switched memory, plasmablasts, double-negative (DN), and CD5^+^ B cells, has been described in our previous study (6).

### 2.4 Statistical analysis

To match sex and age between survivors and non-survivors, 1:2 propensity score matching was performed using R software (version 4.1.0), with a caliper value of 0.2. Normally distributed data are presented as mean ± standard deviation and were compared using the independent-samples t-test. Non-normally distributed data are presented as median (interquartile range) and were compared using the Mann–Whitney test or Friedman test followed by Dunn’s post-test (with *P*-values adjusted using the Bonferroni correction). Categorical variables are presented as numbers (percentages) and were compared using the chi-square or Fisher’s exact test. Spearman’s partial correlation analysis was used to assess the association between Tfh/Tfr cells and B cell subsets as well as clinical parameters, and all correlation *P*-values were adjusted for multiple comparisons using the Benjamini–Hochberg false discovery rate method, with adjusted *P* < 0.05 considered statistically significant. Receiver operating characteristic (ROC) curve analysis was performed to identify the optimal cut-off value by maximizing the Youden index. Conditional logistic regression analysis was performed to evaluate independent predictors, and variables with *P* < 0.05 in univariate analysis were included in the multivariable model. Results are reported as odds ratios (ORs) with 95% confidence intervals (CIs). Statistical significance was set at *P* < 0.05. Multicollinearity was assessed using the variance inflation factor, with values < 10 indicating no significant multicollinearity. All statistical analyses were performed using IBM SPSS Statistics (version 27.0; IBM Corp., Armonk, NY, USA), and graphs were generated using GraphPad Prism 8.0 (GraphPad Software, San Diego, CA, USA).

## 3 Results

### 3.1 Clinical characteristics of the study population

A total of 245 patients with sepsis were included, with a 28-day mortality rate of 17.14% (n = 42); 7.35% (n = 18) died within seven days. As shown in **Supplementary Table 4**, survivors were significantly younger than non-survivors. After propensity score matching for age and sex, the final analysis cohort included 81 survivors and 42 non-survivors (**Supplementary Figure 5**). The demographic and clinical characteristics of the participants are presented in **Table 1**. Non-survivors had significantly higher Lac and C-reactive protein (CRP) levels, higher APACHE II and SOFA scores, and a greater proportion of septic shock. Pulmonary infection was the most common in non-survivors, whereas intra-abdominal and urinary tract infections were more common in survivors than in non-survivors.

**Table 1:** Demographic and clinical data of the study population.

Parameter	Healthy controls (n = 69)	Survivors (n = 81)	Non-survivors (n = 42)	*P* value
Age, years	78.00 (74.00–84.00)	82.00 (76.00–86.00)	82.50 (76.00–87.00)	0.85
Males (n, %)	40 (57.97%)	47 (58.02%)	26 (61.90%)	0.678
Laboratory test				
WBC (10^9^/L)	5.55 (4.71–6.27)	11.92 (7.52–15.05)	9.21 (6.47–16.61)	0.86
Lymphocyte (10^9^/L)	1.62 (1.34–2.16)	0.55 (0.33–0.78)	0.56 (0.30–0.80)	0.898
CD4^+^ T (10^9^/L)	0.57 (0.45–0.73)	0.12 (0.08–0.22)	0.14 (0.10–0.24)	0.376
Lac (mmol/L)		2.40 (1.40–3.50)	3.30 (1.97–4.75)	**0.013**
PCT (ng/mL)		9.88 (1.62–40.42)	14.81 (4.49–76.53)	0.139
CRP (mg/L)		120.78 (48.68–183.12)	160.10 (96.07–212.81)	**0.022**
APACHE II score		13.00 (10.00–16.50)	22.00 (15.00–26.25)	**<0.001**
SOFA		7.00 (4.00–10.00)	11.00 (8.00–15.00)	**<0.001**
Diagnostic (n, %)				
Sepsis		45 (55.56%)	9 (21.43%)	**<0.001**
Septic shock		36 (44.44%)	33 (78.57%)	
Comorbidities (n, %)				
Coronary atherosclerotic heart disease		15 (18.52%)	8 (19.05%)	0.94
Diabetes mellitus		15 (18.52%)	8 (19.05%)	0.94
Hypertension		49 (60.50%)	19 (45.24%)	0.107
Pathogens (n, %)				
Bacteria		43 (53.09%)	16 (38.10%)	0.33
Fungus		1 (1.23%)	2 (4.76%)	
Bacteria and fungi		2 (2.47%)	1 (2.38%)	
Unknown		35 (43.21%)	23 (54.76%)	
Site of infection				
Lungs		22 (27.16%)	24 (57.14%)	**0.001**
Intra-abdominal		28 (34.57%)	8 (19.05%)	
Urinary tract		18 (22.22%)	1 (2.38%)	
Skin soft tissue		4 (4.94%)	1 (2.38%)	
Others		9 (11.11%)	8 (19.05%)	

WBC, white blood cell; Lac, Lactate; PCT, procalcitonin; CRP, C-reactive protein; APACHE, Acute Physiology and Chronic Health Evaluation; SOFA, Sequential Organ Failure Assessment.

Bold *P* values indicate statistical significance (*P* < 0.05).

### 3.2 Tfh subset and Tfr cell depletion with increased Tfh/Tfr ratio at admission

Compared with that in HCs, the overall frequency of Tfh cells did not differ significantly; however, the subset composition was altered. The proportion of Tfh17.1 was significantly reduced, whereas that of Tfh17 cells was significantly increased. In addition, the frequency of Tfr cells was significantly lower in the sepsis group. The absolute counts of Tfh cell subsets, as well as Tfr cells, were significantly decreased in patients with sepsis (*P* < 0.001), whereas the Tfh/Tfr ratio was significantly increased [6.98 (5.52–9.74) vs. 5.52 (4.06–7.56), *P* = 0.002, **Table 2**]. Using the HC median as a baseline, we calculated the relative percentage decrease in CD4^+^ T cells, Tfh, and Tfr in sepsis with the formula: [(median of HCs − patient value)/median of HCs] × 100%, where a larger value indicates a greater decrease. The degree of decrease did not differ between CD4^+^ T cells and Tfh (*P* = 0.378). However, degree of decrease in Tfr was significantly greater than that in CD4^+^ T cells [83.13% (69.16%–90.16%) vs. 76.79% (60.92%–85.19%), *P* < 0.001] and Tfh [83.13% (69.16%–90.16%) vs. 76.53% (57.94%–85.69%), *P* < 0.001].

**Table 2:** Tfh subset percentages and counts, Tfr cells, and Tfh/Tfr ratios at admission.

Parameter	Healthy controls (n = 69)	Patients at admission (n = 123)	*P* value
Tfh, % of CD4^+^T	16.42% (12.24%, 22.59%)	16.42% (11.93%, 19.88%)	0.211
Tfh1, % of Tfh	22.03% ± 7.31%	19.84% ± 8.78%	0.079
Tfh2, % of Tfh	17.73% (13.40%, 20.88%)	17.78% (14.06%, 24.97%)	0.187
Tfh17, % of Tfh	35.58% (30.82%, 41.98%)	38.90% (32.52%, 45.47%)	**0.029**
Tfh17.1, % of Tfh	23.98% (19.52%, 27.81%)	20.19% (14.57%, 25.16%)	**<0.001**
Tfr, % of CD4^+^T	2.58% (1.87%, 3.04%)	1.71% (1.19%, 2.45%)	**<0.001**
Tfh (10^6^/L)	86.38 (68.56, 131.43)	20.27 (12.37, 36.33)	**<0.001**
Tfh1 (10^6^/L)	19.05 (13.67, 25.38)	3.46 (1.92, 6.84)	**<0.001**
Tfh2 (10^6^/L)	15.16 (11.23, 20.79)	3.76 (1.87, 7.04)	**<0.001**
Tfh17 (10^6^/L)	32.35 (23.02, 44.07)	7.65 (5.02, 14.39)	**<0.001**
Tfh17.1 (10^6^/L)	19.68 (14.84, 34.34)	3.44 (1.95, 7.79)	**<0.001**
Tfr (10^6^/L)	13.74 (9.57, 17.77)	2.32 (1.35, 4.24)	**<0.001**
Tfh/Tfr	5.52 (4.06, 7.56)	6.98 (5.52, 9.74)	**0.002**

Tfh, follicular helper T cells; Tfr, follicular regulatory T cells.

Bold *P* values indicate statistical significance (*P* < 0.05).

### 3.3 Tfh subsets and Tfr cells are associated with prognosis

On day 1, the proportion of Tfh cells was significantly lower in non-survivors than in survivors (14.22% [10.41%–16.99%] vs. 17.36% [12.16%–20.25%], *P* = 0.024; **Figure 1A**). However, no significant difference in absolute Tfh counts was observed between the two groups (**Figure 2A**), possibly due to higher median CD4^+^ T cell counts in non-survivors. No significant differences were observed in the proportions or absolute counts of Tfh1, Tfh2, Tfh17, Tfh17.1, or Tfr cells, nor in the Tfh/Tfr ratio (**Figures 1B, C**, **2B–D**). After excluding patients with no day 7 sample because of transfer, death, treatment withdrawal, or for other reasons, 84 patients remained for analysis on day 7, including 66 survivors and 18 non-survivors. On day 7, the reduction in Tfr cell percentage was borderline significant (1.55% [1.06%–1.93%] vs. 1.94% [1.24%–2.91%], *P* = 0.05; **Figure 1F**). No significant differences were observed in the proportions of Tfh cell subsets between the two groups (**Figures 1D, E**). Absolute counts were significantly lower in non-survivors than in survivors for Tfh (29.28 [20.01–59.99] vs. 65.45 [47.57–112.73]), Tfh1 (5.69 [2.30–14.31] vs. 11.71 [6.61–21.91]), Tfh2 (6.49 [3.88–12.01] vs. 9.81 [7.01–18.45]), Tfh17 (12.10 [8.47–19.40] vs. 24.94 [15.91–44.19]), Tfh17.1 (6.08 [2.73–14.90] vs. 16.05 [9.40–28.53]), and Tfr (2.07 [1.43–10.70] vs. 8.58 [3.71–12.73]) (**Figures 2E–G**). Absolute lymphocyte and CD4^+^ T cell counts were also significantly lower in non-survivors than in survivors on day 7 (**Supplementary Table 5**). No significant difference in the Tfh/Tfr ratio was observed between survivors and non-survivors on day 7 (**Figure 2H**).

**Figure 1:**
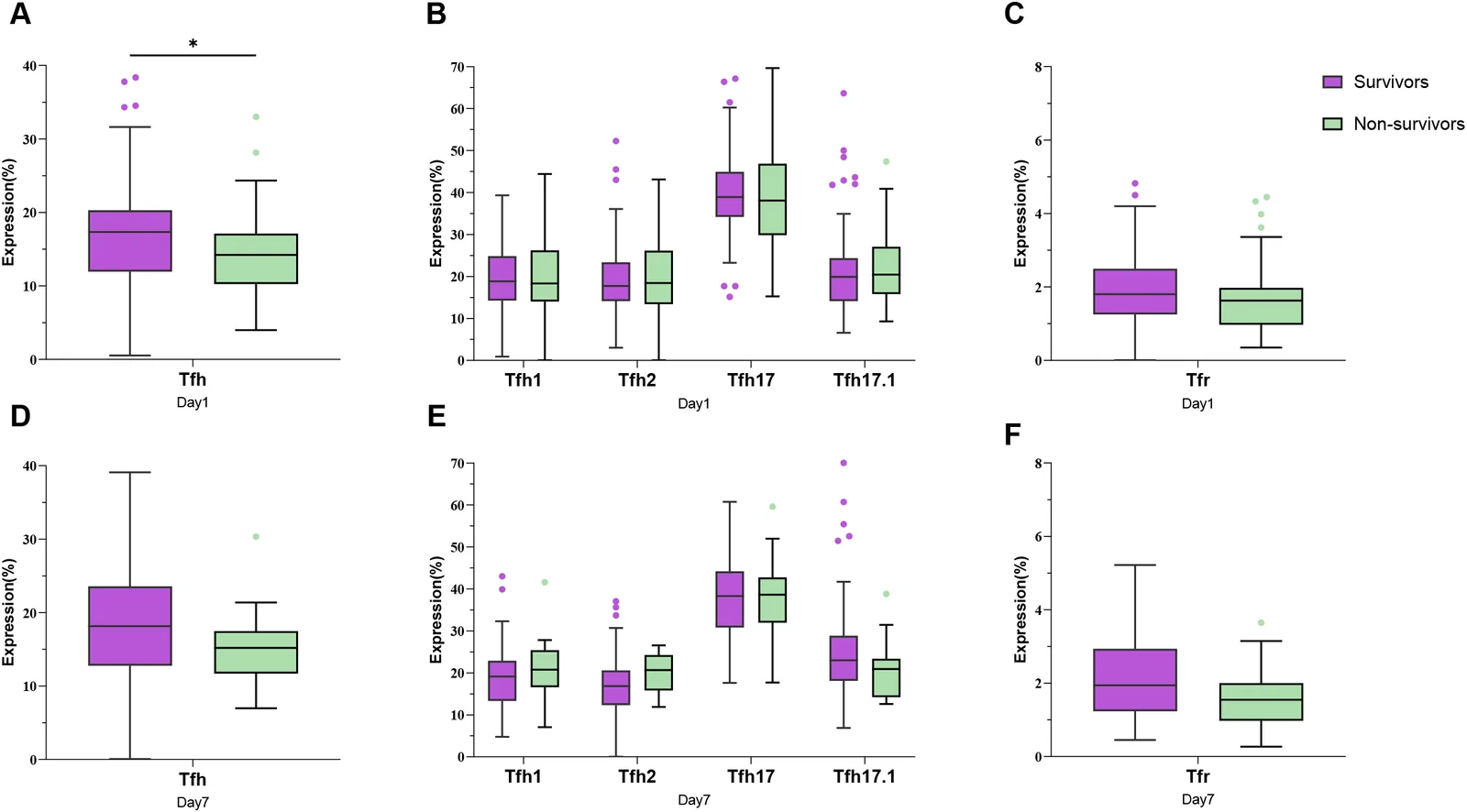
Proportions of follicular helper T (Tfh) **(A)**, Tfh1, Tfh2, Tfh17, and Tfh17.1 **(B)**, and follicular regulatory T (Tfr) **(C)** in sepsis survivors and non-survivors on day 1. Proportions of Tfh **(D)**, Tfh1, Tfh2, Tfh17, and Tfh17.1 **(E)**, and Tfr **(F)** in sepsis survivors and non-survivors on day 7. Lines within boxes represent median values. The upper and lower edges of the box represent the 75th and 25th percentiles, respectively, and whiskers extend to 1.5 times the interquartile range. Statistical significance was set at *P* < 0.05 (*P < 0.05).

**Figure 2:**
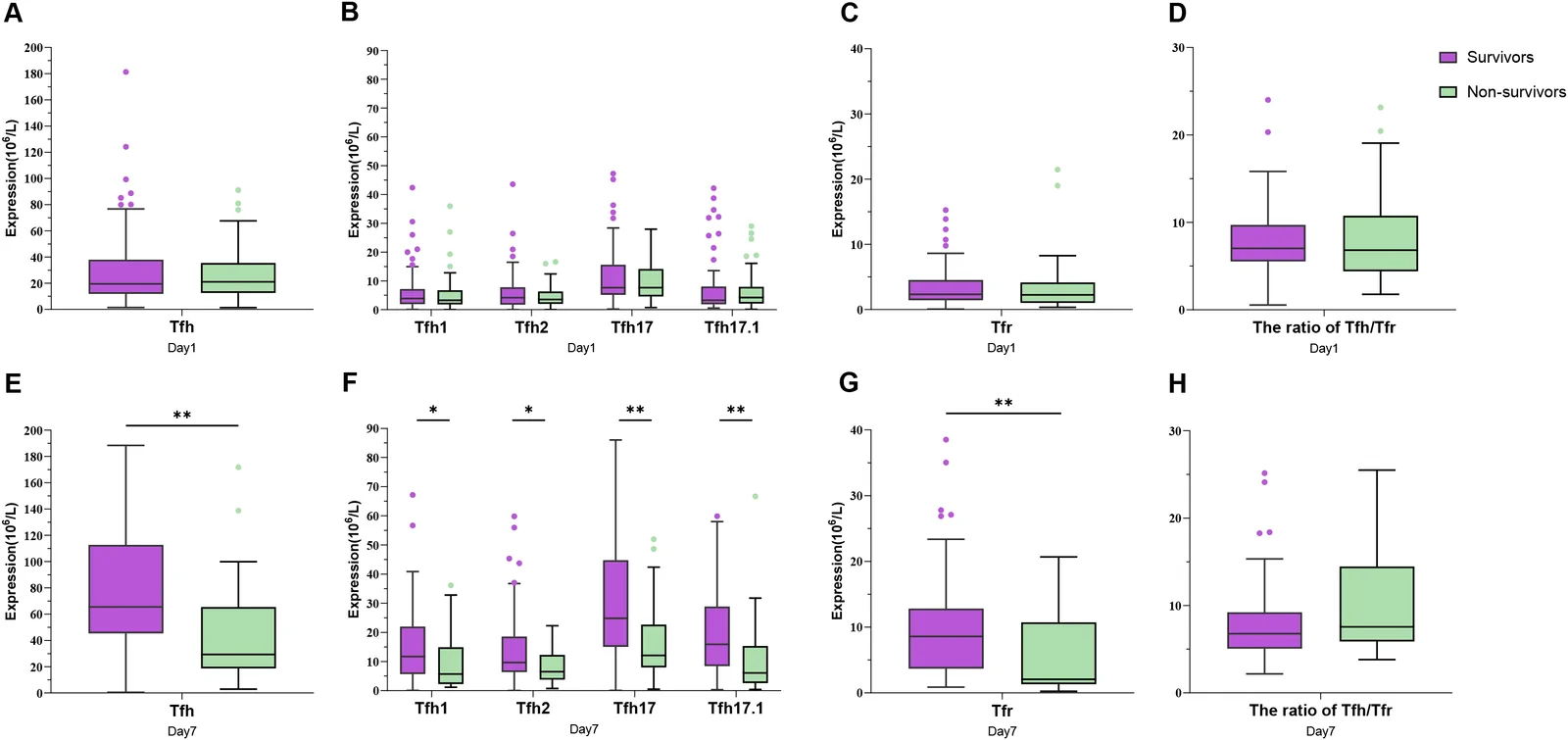
Absolute counts of follicular helper T (Tfh) **(A)**, Tfh1, Tfh2, Tfh17, and Tfh17.1 **(B)**, follicular regulatory T (Tfr) **(C)**, and the Tfh/Tfr ratio **(D)** in sepsis survivors and non-survivors on day 1. Absolute counts of Tfh **(E)**, Tfh1, Tfh2, Tfh17, and Tfh17.1 **(F)**, Tfr **(G)**, and the Tfh/Tfr ratio **(H)** in sepsis survivors and non-survivors on day 7. Lines within boxes represent median values. The upper and lower edges of the box represent the 75th and 25th percentiles, respectively, and whiskers extend to 1.5 times the interquartile range. Statistical significance was set at *P* < 0.05 (*P < 0.05, **P < 0.01).

As **Table 3** shows, ROC curve analysis was used to determine the optimal cutoff value for Tfh proportion at admission, followed by conditional logistic regression analysis to evaluate its prognostic value for 28-day mortality in patients with sepsis. Other covariates included CRP level, APACHE II score, SOFA score, Lac level, and site of infection (lung or other sites). Univariate analysis indicated that all these variables were associated with 28-day mortality. Variance inflation factor analysis showed no evidence of multicollinearity. Multivariate analysis identified APACHE II score and pulmonary infection as independent risk factors, whereas Tfh (%) ≥ 17.05% was independently associated with lower 28-day mortality in patients with sepsis (OR [95% CI]: 0.331 [0.127, 0.861], *P* = 0.023).

**Table 3:** Univariate and multivariate conditional logistic regression analyses of mortality in patients with sepsis.

Variable	Univariate analysis		Multivariate analysis	
	OR (95% CI)	*P* value	OR (95% CI)	*P* value
CRP	1.005 (1.000, 1.010)	0.03		NS
APACHE II	1.114 (1.049, 1.183)	<0.001	1.109 (1.037, 1.185)	0.003
SOFA	1.209 (1.091, 1.340)	<0.001		NS
Lac	1.203 (1.034, 1.399)	0.017		NS
Tfh (%) ≥ 17.05%**^a^**	0.333 (0.155, 0.718)	0.005	0.331 (0.127, 0.861)	0.023
Lung/other sites	4.165 (1.718, 10.102)	0.002	4.348 (1.557, 12.143)	0.005

OR, odds ratio; CI, confidence interval; CRP, C-reactive protein; APACHE, Acute Physiology and Chronic Health Evaluation; SOFA, Sequential Organ Failure Assessment; Lac, Lactate; Tfh, follicular helper T cells; NS, not significant; ROC, Receiver operating characteristic; AUC, area under the curve.

**^a^**Optimal cutoff for Tfh percentage was determined using an ROC curve: 17.05% (AUC = 0.625, 95% CI = 0.523–0.726, *P* = 0.024; sensitivity = 54.3%, specificity = 76.2%).

### 3.4 Correlation of Tfh and Tfr cells with B cell subsets and clinical parameters

Peripheral blood B cell subsets (Tr, naïve, unswitched memory, switched memory, plasmablasts, DN, and CD5^+^ B cells) were simultaneously measured in patients with sepsis. These data have been previously reported (6). Compared with that in HCs, the absolute count of DN B cells in patients with sepsis showed a decreasing trend, whereas other B cell subsets were significantly reduced (6). Additionally, a sustained decline in Tr B and CD5^+^ B cells was associated with poor prognosis (6). Correlation analysis was performed to better understand the Tfh-Tfr-B cell axis in sepsis. Because lymphopenia may confound these correlations, Spearman’s partial correlation analysis was used, adjusting for absolute total lymphocyte count. On day 1, Tfh, Tfh17, Tfh17.1, and Tfr cells positively correlated with unswitched memory, switched memory, and CD5^+^ B cells. Tfh2 positively correlated with switched memory B cells (**Supplementary Table 6**). Tfh cell subsets and Tfr cells showed no significant correlation with Lac, PCT, CRP, APACHE II score, or SOFA score (**Supplementary Table 7**).

## 4 Discussion

Sepsis is characterized by severe immune dysregulation, and immune function is a key determinant of its prognosis (35). In this study, we analyzed Tfh subsets and Tfr cells in the peripheral blood of patients with sepsis and investigated their associations with B cell subsets and 28-day mortality. Our results showed that Tfh subset composition was altered and Tfh/Tfr ratio was elevated in sepsis. Notably, non-survivors had a lower Tfh cell percentage than survivors, and Tfh (%) ≥ 17.05% was independently associated with lower 28-day mortality in sepsis.

Tfh cells are indispensable for sustained and efficient humoral immunity, as they promote B cell maturation and support the formation of long-lived, high-affinity antibody-secreting cells and memory B cells (36). Tfh cell frequencies did not differ significantly between patients with sepsis and HCs, whereas their absolute counts were significantly decreased. The degree of reduction did not differ between CD4^+^ T cells and Tfh in patients with sepsis. These findings indicate that the decline in Tfh cell counts in sepsis predominantly reflects CD4^+^ T lymphocytopenia rather than specific depletion. Lower Tfh counts in non-survivors on day 7 primarily reflect differences in recovery from CD4^+^ T lymphocytopenia, but they may nevertheless impair maintenance of the GC response.

When percentages were considered, non-survivors had a markedly lower Tfh cell percentage than survivors on day 1; this result is consistent with the findings of Duan et al. (7). Tfh percentage ≥ 17.05% was independently associated with lower 28-day mortality, suggesting that in the context of lymphopenia, the relative preservation of Tfh cells is beneficial. This highlights that compartmental imbalance among helper T cells is as critical to immune dysregulation in sepsis as the absolute quantitative changes in subsets (37, 38).

Tfh and Tfr cells positively correlated with CD5^+^ and memory B cell subsets. B cells acting as key antigen-presenting cells initiate Tfh cell differentiation (39), and Tfh cells in turn promote the development of antibody-secreting cells or memory B cells (13), which may partially account for these findings. The positive correlation between Tfr cells and B cell subsets may reflect coordinated expansion within the Tfh-Tfr axis to maintain immune balance. Interestingly, we found no correlation between Tfh subsets and plasmablasts. One possible explanation is that plasmablasts can be generated through non-GC pathways (40), and these cells are the main source of antibodies in the early stages of infection (41). Overall, the functional relationship within the Tfh-Tfr-B cell axis in sepsis remains to be further investigated.

Our study showed an altered circulating Tfh subset composition in patients with sepsis: Tfh17.1 decreased significantly, whereas Tfh17 appeared relatively increased, despite significant reductions in the absolute counts of all Tfh subsets compared with HCs. However, the percentages of Tfh subsets did not differ significantly between survivors and non-survivors, suggesting that the composition of the Tfh compartment may not determine clinical outcomes.

Reduction in Tfr cells, unlike that in Tfh cells, may have exceeded CD4^+^ T cell reduction in patients with sepsis. The Tfh/Tfr ratio was also markedly elevated, possibly due to a disproportionate reduction in Tfr cells. In patients with COVID-19 and sepsis, Tfr and precursor Tfr are significantly reduced, and this reduction is associated with an increase in anti-interferon-γ autoantibodies and activated atypical B cells (42). The balance between Tfh and Tfr cells is crucial for promoting high-affinity clone selection and constraining autoreactive B cells (32). Therefore, the mechanisms underlying the increased Tfh/Tfr ratio, and whether these changes are associated with immune dysfunction in sepsis, warrant further investigation.

This study has some limitations. First, this was a single-center study with a relatively homogeneous population, and the cutoff of 17.05% was derived and tested in the same cohort. Therefore, it should be considered exploratory, and validation in an independent cohort is needed. Second, the day 7 analysis is subject to survivor bias and non-random case attrition; therefore, the association between day 7 immune parameters and mortality cannot be extrapolated to all patients with sepsis. Third, we assessed only the number and frequency of cell subsets, and correlation alone cannot establish a functional interaction or demonstrate causality; dedicated functional studies are therefore needed. Finally, measurements at only two time points may not fully capture the dynamic trajectory of immune responses during disease progression. Therefore, future studies should include longitudinal assessments with more frequent sampling.

In conclusion, Tfh cell subsets in sepsis are redistributed, with an elevated Tfh/Tfr ratio. A Tfh (%) ≥ 17.05% was independently associated with lower 28-day mortality. These findings provide new insights into immune dysregulation in sepsis.

## Data Availability

The raw data supporting the conclusions of this article will be made available by the authors, without undue reservation.
