# Supplementary material for: Circulating follicular helper T cells are associated with mortality in sepsis
Source: Front Immunol. 2026 Oct 28;17:1931433. doi: 10.3389/fimmu.2026.1931433 (PMC13751345; doi:10.3389/fimmu.2026.1931433)
Supplement: Supplementary file 1 [file SupplementaryFile1.docx]

Supplementary Material

# 1 Supplementary Figures and Tables

## 1.1 Supplementary Figures

**Supplementary Figure 1.** Immune cell staining performed by flow cytometry

**Supplementary Figure 2.** Gating strategies for identifying circulating follicular helper T (Tfh) cell subsets

**Supplementary Figure 3.** Gating strategies for identifying circulating follicular regulatory T (Tfr) cells and the Tfh/Tfr ratios

**Supplementary Figure 4.** Gating strategies for identifying circulating T lymphocytes and CD4^+^ T cells

**Supplementary Figure 5 .** Screening process of the study population

## 1.2 Supplementary Tables

**Supplementary Table 1.** Staining panel for monoclonal fluorescent antibody labeling of circulating Tfh cell subsets.

| **Fluorochrome** | **Marker** | **Clone** | **Source** |
| --- | --- | --- | --- |
| KO | CD45 | J.33 | Beckman Coulter |
| PE | CD4 | 13B8.2 | Beckman Coulter |
| APC | CCR7 | G043H7 | BioLegend |
| ECD | CD45RO | UCHL1 | Beckman Coulter |
| PC7 | CCR6 | G034E3 | BioLegend |
| BV421 | CXCR5 | J252D4 | BioLegend |
| FITC | CXCR3 | G025H7 | BioLegend |

**Supplementary Table 2.** Staining panel for monoclonal fluorescent antibody labeling of circulating follicular regulatory T (Tfr) cells and Tfh/Tfr ratios.

| **Fluorochrome** | **Marker** | **Clone** | **Source** |
| --- | --- | --- | --- |
| KO | CD45 | J.33 | Beckman Coulter |
| AA750 | CD4 | 13B8.2 | Beckman Coulter |
| FITC | CD45RA | ALB11 | Beckman Coulter |
| BV421 | CXCR5 | J252D4 | BioLegend |
| PE | CD25 | B1.49.9 | Beckman Coulter |
| PC7 | CD127 | R34.34 | Beckman Coulter |

KO, Krome Orange; PE, Phycoerythrin; APC, Allophycocyanin; ECD, Phycoerythrin-Texas Red; PC7, Phycoerythrin-Cyanin 7; BV421, Brilliant Violet 421; FITC, Fluorescein isothiocyanate; AA750, APC-Alexa Fluor 750.

**Supplementary Table 3.** Staining panel for monoclonal fluorescent antibody labeling of circulating T lymphocytes and CD4^+^ T cells.

| **Fluorochrome** | **Marker** | **Clone** | **Source** |
| --- | --- | --- | --- |
| KO | CD45 | J.33 | Beckman Coulter |
| AA750 | CD3 | UCHT1 | Beckman Coulter |
| PE | CD4 | 13B8.2 | Beckman Coulter |
| AA700 | CD8 | B9.11 | Beckman Coulter |

KO, Krome Orange; AA750,APC- Alexa Fluor 750; PE, Phycoerythrin; AA700, APC-Alexa Fluor 700.

**Supplementary Table 4.** Baseline characteristics of patients with sepsis.

| **Parameter** | **Survivors**  **(n = 203)** | **Non-survivors**  **(n = 42)** | ***P***  **value** |
| --- | --- | --- | --- |
| Age, years | 74.00 (61.00–83.00) | 82.50 (76.00–87.00) | <0.001 |
| Males (n, %) | 120 (59.11%) | 26 (61.90%) | 0.737 |

**Supplementary Table 5.** White blood cell, total lymphocyte, and CD4^+^ T cell counts on day 7 in patients with sepsis

| **Parameter** | **Survivors**  **(n = 66)** | **Non-survivors**  **(n = 18)** | ***P* value** |
| --- | --- | --- | --- |
| WBC (10^9^/L) | 7.92 (5.42–9.75) | 8.75 (6.23–16.04) | 0.088 |
| Lymphocyte (10^9^/L) | 0.99 (0.74–1.41) | 0.63 (0.33–1.15) | **<0.001** |
| CD4^+^ T (10^9^/L) | 0.39 (0.28–0.60) | 0.24 (0.11–0.36) | **0.011** |

WBC, white blood cell.

Bold values indicate statistical significance (*P* < 0.05).

**Supplementary Table 6.** Correlation of follicular helper T (Tfh) cell subsets, follicular regulatory T (Tfr) cells, and B cell subsets on day 1 in sepsis.

|  |  | B cells (10^6^/L) | Transitional B (10^6^/L) | Naïve B (10^6^/L) | Unswitched memory B (10^6^/L) | Switched memory B (10^6^/L) | Plasmablasts (10^6^/L) | DN B (10^6^/L) | CD5^+^ B (10^6^/L) |
| --- | --- | --- | --- | --- | --- | --- | --- | --- | --- |
| Tfh (10^6^/L) | *r* | 0.171 | 0.079 | 0.138 | **0.231** | **0.334** | 0.11 | 0.114 | **0.284** |
|  | *P* | 0.193 | 0.567 | 0.270 | **0.047** | **0.014** | 0.397 | 0.385 | **0.019** |
| Tfh1 (10^6^/L) | *r* | 0.014 | 0.073 | 0.019 | -0.033 | 0.213 | 0.022 | 0.092 | 0.141 |
|  | *P* | 0.911 | 0.572 | 0.883 | 0.804 | 0.072 | 0.870 | 0.506 | 0.263 |
| Tfh2 (10^6^/L) | *r* | 0.141 | 0.074 | 0.154 | 0.1 | **0.252** | 0.072 | 0.099 | 0.121 |
|  | *P* | 0.263 | 0.572 | 0.219 | 0.460 | **0.031** | 0.572 | 0.460 | 0.345 |
| Tfh17 (10^6^/L) | *r* | 0.181 | 0.065 | 0.135 | **0.316** | **0.319** | 0.17 | 0.085 | **0.251** |
|  | *P* | 0.161 | 0.623 | 0.274 | **0.014** | **0.014** | 0.193 | 0.530 | **0.031** |
| Tfh17.1 (10^6^/L) | *r* | 0.16 | 0.058 | 0.123 | **0.229** | **0.243** | -0.001 | 0.088 | **0.318** |
|  | *P* | 0.218 | 0.653 | 0.342 | **0.047** | **0.036** | 0.994 | 0.520 | **0.014** |
| Tfr (10^6^/L) | *r* | 0.156 | 0.053 | 0.163 | **0.263** | **0.274** | -0.023 | -0.036 | **0.25** |
|  | *P* | 0.219 | 0.685 | 0.212 | **0.024** | **0.019** | 0.870 | 0.804 | **0.034** |
| Tfh/Tfr | *r* | 0.008 | -0.049 | -0.062 | -0.034 | 0.15 | 0.201 | 0.154 | -0.075 |
|  | *P* | 0.948 | 0.704 | 0.638 | 0.804 | 0.231 | 0.097 | 0.219 | 0.572 |

DN, double-negative.

Bold values indicate statistical significance (*P* < 0.05).

**Supplementary Table 7.** Correlation of follicular helper T (Tfh) cell subsets, follicular regulatory T (Tfr) cells, and clinical parameters on day 1 in sepsis.

|  |  | Lactate  (mmol/L) | PCT  (ng/mL) | CRP  (mg/L) | APACHE II score | SOFA |
| --- | --- | --- | --- | --- | --- | --- |
| Tfh (10^6^/L) | *r* | -0.138 | 0.002 | 0.055 | 0.009 | 0.024 |
|  | *P* | 0.462 | 0.986 | 0.761 | 0.952 | 0.869 |
| Tfh1 (10^6^/L) | *r* | -0.088 | -0.072 | -0.137 | -0.105 | -0.086 |
|  | *P* | 0.710 | 0.761 | 0.462 | 0.705 | 0.710 |
| Tfh2 (10^6^/L) | *r* | -0.021 | 0.114 | 0.067 | 0.047 | 0.09 |
|  | *P* | 0.869 | 0.668 | 0.761 | 0.761 | 0.710 |
| Tfh17 (10^6^/L) | *r* | -0.142 | 0.024 | 0.165 | 0.036 | 0.049 |
|  | *P* | 0.462 | 0.869 | 0.408 | 0.839 | 0.761 |
| Tfh17.1 (10^6^/L) | *r* | -0.151 | -0.054 | -0.055 | 0.052 | 0.029 |
|  | *P* | 0.462 | 0.761 | 0.761 | 0.761 | 0.869 |
| Tfr (10^6^/L) | *r* | -0.228 | -0.056 | -0.102 | -0.165 | -0.067 |
|  | *P* | 0.408 | 0.761 | 0.705 | 0.408 | 0.761 |
| Tfh/Tfr | *r* | 0.172 | 0.098 | 0.193 | 0.166 | 0.074 |
|  | *P* | 0.408 | 0.707 | 0.408 | 0.408 | 0.761 |

PCT, procalcitonin; CRP, C-reactive protein; APACHE, Acute Physiology and Chronic Health Evaluation; SOFA, Sequential Organ Failure Assessment.
